# Soluble 4R0N Tau Abrogates Endocytic Vesicular Dynamics

**DOI:** 10.3389/fnagi.2020.537712

**Published:** 2020-11-05

**Authors:** Tharun Selvam Mahendran, S. N. Suresh, Lakshmi Garimella, Ravi Manjithaya

**Affiliations:** ^1^Molecular Biology and Genetics Unit, Jawaharlal Nehru Centre for Advanced Scientific Research, Bangalore, India; ^2^Centre for Brain Research, Indian Institute of Science, Bangalore, India; ^3^Neuroscience Unit, Jawaharlal Nehru Centre for Advanced Scientific Research, Bangalore, India

**Keywords:** Alzheimer’s disease, vesicular trafficking, amyloid precursor protein (APP), endosome dysfunction, autophagic stress, soluble 4R0N tau, endocytic dysfunction, vesicle tracking

## Abstract

Aggregated tau is a hallmark neuropathological feature in numerous neurodegenerative disorders. Previous studies aiming to validate aggregated tau pathology as a pathogenic driver of neurodegeneration in correlation to characteristic behavioral phenotypes have had shortcomings. Although studies on soluble tau pathology have effectively addressed these shortcomings, the role of soluble tau in the molecular pathogenesis of neurodegeneration is not yet unequivocally established. In sporadic Alzheimer’s disease (AD), the relevance of soluble tau pathology in endolysosomal dysfunction and autophagic stress, some of the earliest disease manifestations, is unclear. In this study, we report that soluble 4R0N tau overexpression affects the expression levels of certain markers associated with the endolysosomal system and autophagy. Moreover, through live-cell imaging, we found that the vesicular dynamics of early endosomes were affected with respect to spatiotemporal parameters and vesicle maturation. Additionally, we observed the localization of amyloid precursor protein (APP) along the endocytic pathway and found that upon overexpression of soluble 4R0N tau, APP was preferentially localized to the endocytic compartments implicated in the amyloidogenic pathway. Overall, our observations indicate that soluble 4R0N tau abrogates the dynamics of the endolysosomal system, autophagy, and affects the trafficking of APP. Since the amyloidogenic processing of APP occurs during its progression through the endocytic pathway, our results suggest that the generation of amyloid-β (Aβ) might also be modulated.

## Introduction

Tau is an intrinsically disordered proteinaceous cytoskeletal element known to associate with microtubules and bundle actin filaments (Weingarten et al., 1975; He et al., 2009; Morris et al., 2011), and it is highly expressed in the human central nervous system as six major isoforms *via* alternative splicing (Mandelkow and Mandelkow, 2012). In a physiological context, tau remains soluble and functional, but in the case of idiopathic neurodegenerative disorders such as sporadic Alzheimer’s disease (AD), elevated levels of tau (Khatoon et al., 1992) become highly susceptible to conformational changes, aggregation, and oligomerization and further assembly into paired helical filaments (PHFs), which then nucleate into neurofibrillary tangles (NFTs; Wille et al., 1992; Crowther et al., 1994; Wilson and Binder, 1995; Patterson et al., 2011). For simplicity, we shall refer to pathological tau conformers including oligomers, PHFs, and NFTs as tau aggregates. Gain-of-function toxicity of tau has been implicated in the pathogenesis of AD, which is predominantly characterized by the presence of pathological protein aggregates such as tau aggregates and amyloid-β (Aβ) containing senile plaques (Mitchell et al., 2002; Maeda et al., 2006; Johnson et al., 2016; Schöll et al., 2016). This toxicity was presumably attributed to aggregated tau formed by the nucleation events involving soluble tau, mediated by liquid–liquid phase separation (Ambadipudi et al., 2017; Wegmann et al., 2018). However, there is a lack of evidence to unequivocally establish which aggregated state(s) of tau has a role in the cascade of events leading to neurodegeneration.

The presence of aggregated tau in the brains of transgenic mice was found to be correlated with behavioral and electrophysiological abnormalities as well as neuronal loss, characteristic of clinical AD pathology. Upon closer analysis, when the expression level of tau, that is yet to aggregate, was reduced later in the lifespan of these mice, some of the initially observed AD pathology-related phenotypes were ameliorated despite the prevalence of tau aggregates (Santacruz et al., 2005). These observations imply that tau in a soluble form, rather than the end-point aggregated form, is responsible for AD pathology-related phenotypes. In accordance with the previous observations, several studies have revealed that soluble tau is responsible for neuronal loss through the activation of caspases as well as synaptic dysfunction that underlies cognitive impairment (Spires-Jones et al., 2008; de Calignon et al., 2010; Bolós et al., 2017). Furthermore, transgenic mice engineered to overexpress tau with a high propensity to aggregate, do not successfully mirror behavioral and histopathological phenotypes observed in AD-related clinical studies (McGowan et al., 2006; Dawson et al., 2018; Strang et al., 2018).

The pathological relevance of soluble tau in AD pathology has been demonstrated by multiple studies, as mentioned earlier. However, the role of soluble tau in the pathogenesis of AD is still unclear. Many studies have reported correlations between pathologies of soluble tau and Aβ thereby hinting toward possible crosstalk. In the case of neuropathology, accumulation of soluble tau has been shown to precede that of Aβ plaque deposition, and it also correlates well with aging (Braak et al., 2011). A study involving electrophysiology showed that soluble tau-induced silencing of neuronal activity overshadowed Aβ-induced neuronal hyperactivity, indicating that tau pathology was more impactful than that of Aβ (Busche et al., 2019). Moreover, soluble tau pathology exacerbates Aβ-induced neurotoxicity in AD-related transgenic mice (Roberson et al., 2007; Ittner et al., 2010). More direct correlations between soluble tau and Aβ pathologies have also been demonstrated in recent years. Soluble tau pathology can potentially drive Aβ production directly through the STAT1–BACE1 axis (Zhang et al., 2018). Passive immunization of AD transgenic mice with antibodies against tau, resulted in a reduction in the total Aβ burden, indicating a potential cross-talk between soluble tau and the amyloidogenic pathway, which gives rise to Aβ (Dai et al., 2017). Collectively, these studies hint that tau could be playing an upstream role relative to Aβ in the progression of AD pathogenesis.

However, a direct connection between soluble tau pathology and the production of Aβ is lacking. Since Aβ generation primarily occurs at the endocytic compartments due to the interaction between amyloid-β precursor protein (APP) and β-secretase (BACE1; Rajendran et al., 2006; Das et al., 2015), we hypothesized that soluble tau pathology could contribute to Aβ generation through disruption of endocytic vesicular dynamics. Hence, we designed this study to elucidate the effect of soluble tau on endocytic vesicular dynamics and trafficking of APP along the endocytic pathway. Such an endeavor will require close attention to the major compartments of the endolysosomal system such as the early endosomes, late endosomes, and lysosomes labeled with the RAB/LAMP protein markers. As the model for our study, we have used HeLa cells with episomal overexpression of 4R0N tau (soluble 4R0N tau; Hoover et al., 2010), which is an alternatively spliced variant of tau that contains four repeats of the microtubule-binding domain without the N-terminal sequence and contains exons 1, 4 and 5, 7, and 9–13, intron 13, and exon 14. The endolysosomal system of HeLa does not operate in a complex regulatory manner such as that observed in certain specialized cell types with very dynamic membrane/vesicle homeostasis, thereby allowing for highly interpretable readouts of the endocytic vesicular dynamics (Parton et al., 1992; Overly and Hollenbeck, 1996; Nixon, 2005). Moreover, HeLa cells do not show a detectable level of tau mRNA transcripts, which minimizes the chances of observing overexpression artifacts during experimentation with the 4R0N tau plasmid construct (Thul et al., 2017). In addition, the relatively symmetric morphology of HeLa cells enables highly robust and reproducible computational analysis of endocytic vesicular dynamics without the need for manual segmentation for different cell regions. For the ease of quantitative analysis and a lesser degree of variability, we opted to use HeLa as the model for our study.

The current study aims to understand the *in cellulo* effect of soluble 4R0N tau overexpression on the endolysosomal system and trafficking of APP as a cargo. In our study, we show that overexpression of soluble 4R0N tau correlates with impaired endolysosomal flux, induction of autophagic stress, and enhanced sequestration of APP into endocytic compartments, which can potentially stimulate the amyloidogenic pathway.

## Materials and Methods

### Chemicals and Antibodies

Anti-rabbit IgG Atto 633 (41176), anti-mouse IgG Atto 633 (78102), and trypsin-EDTA (59418C) were purchased from Sigma–Aldrich (St. Louis, MO, USA). Anti-rabbit IgG antibody conjugated with horseradish peroxidase (7074), anti-β-actin antibody (4970), anti-LAMP1 antibody (9091), anti-RAB5 antibody (3547), and anti-RAB7 antibody (9367) were purchased from Cell Signaling Technology (Danvers, MA, USA). Beclin-1 (sc-48341) antibody was purchased from Santa Cruz Biotechnology (Dallas, TX, USA). Anti-mouse IgG antibody conjugated with horseradish peroxidase (172–1011) was purchased from Bio-Rad (Hercules, CA, USA). Plasmid miniprep kit was purchased from Qiagen (Hilden, Germany).

### Plasmid Constructs

Plasmids used for the experiments include pRK5–EGFP-tau (hereafter 4R0N tau or soluble 4R0N tau; gift from Karen Ashe; Addgene, 46904; Hoover et al., 2010), pEGFP-N1 (hereafter vector control or control; 6085-1, Clontech), mCh-RAB5 (gift from Gia K. Voeltz; Addgene, 49201; Friedman et al., 2010), pmRFP-LC3 (gift from Tamotsu Yoshimori; Addgene, 21075; Kimura et al., 2007), pDsRedm-C1:APP (gift from Prof. Erika Holzbaur, University of Pennsylvania, Philadelphia, PA, USA; Fu and Holzbaur, 2013). All of the plasmid constructs used in the study were validated by DNA sequencing and immunoblot analysis prior to experimentation (data not shown).

### Transfection

HeLa cells were seeded such that the cultured cells reached 50–60% confluency by the following day for transfection. Transfection of HeLa cells with the appropriate plasmids was conducted using the Lipofectamine 2000 transfection reagent (11668-019, Life Technologies, Carlsbad, CA, USA) in accordance with the manufacturer’s instructions. All cell culture-related experiments were performed 48 h after transfection.

### Cell Culture

HeLa cells were maintained in Dulbecco’s modified Eagle medium (DMEM; Sigma–Aldrich, D5648) supplemented with 3.7 g/l of sodium bicarbonate (Sigma–Aldrich, S5761), 10% fetal bovine serum (FBS; PAN, 3302-P121508), and 100 U/ml of penicillin and streptomycin (Sigma–Aldrich, P4333). The cells were maintained at 37°C in a humidified atmosphere with 5% CO_2_. For immunofluorescence experiments, subconfluent HeLa cells were seeded in 60-mm dishes containing coverslips and incubated overnight. Subsequently, cells were transfected with the respective plasmids and incubated for 48 h until they were processed for microscopy. For live-cell imaging-related experiments, subconfluent HeLa cells were seeded in coverslip bottom cell-imaging dishes (0030740017, Eppendorf, Hamburg, Germany). Cells were transfected with the respective plasmids, then incubated for 48 h and imaged. For the immunoblotting experiments, HeLa cells were seeded in six-well dishes and incubated overnight. Cells were then transfected with the respective plasmids, incubated for 48 h, and subsequently processed for immunoblot experiments.

### Immunofluorescence

After 48 h of transfection, HeLa cells seeded on coverslips were washed with 1× PBS, fixed with 4% paraformaldehyde (Sigma–Aldrich, P6148), and then permeabilized with 0.25% Triton X-100 (MB03, HiMedia Laboratories, Mumbai, India) followed by incubation with the respective primary antibody, overnight at 4°C. The primary antibodies were diluted appropriately in 0.25% BSA in 1× PBS. After primary antibody incubation, the coverslips were incubated with the appropriate secondary antibody for 2 h at RT. The appropriate secondary antibody dilutions were made in 0.25% BSA in 1× PBS. The coverslips were mounted on slides using Vectashield mounting medium (H-1,000, Vector Laboratories, Burlingame, CA, USA).

### Immunoblotting

After 48 h of transfection, the cells were washed with 1× PBS, scraped, and collected in standard Laemmli buffer. The lysates were boiled for 10 min at 95°C. The lysates were then subjected to SDS–PAGE and subsequently transferred onto a PVDF (Bio-Rad, 162-0177) membrane using the Transblot Turbo transfer system (Bio-Rad). The blots were stained with Ponceau S to check for efficient protein transfer and were then de-stained using 1× PBS. The blots were then incubated with the appropriate primary antibody dilution, after a blocking step, prepared in 0.25% BSA in 1× PBS, overnight at 4°C. Subsequently, the blots were incubated with the appropriate HRP-conjugated secondary antibody dilution prepared in 5% skim milk powder (GRM1254, HiMedia Laboratories) in 1× PBS, for 1 h at 37°C. The blots were washed with 1× PBS/PBST periodically after each step to remove any residual reagent from prior steps. The blots were developed using an enhanced chemiluminescence substrate (Clarity, Bio-Rad) prior to imaging using a gel documentation system (G-Box, Syngene, UK). β-actin was used as the loading control. Band intensities were calculated using Fiji (Schneider et al., 2012; ImageJ; NIH, Bethesda, MD, USA).

### Microscopy and Vesicle Tracking

For the immunofluorescence experiments, except for the “APP colocalization” experiments, the photomicrographs were acquired using the DeltaVision Elite widefield microscope (GE Healthcare, Chicago, IL, USA) equipped with a 60× oil immersion objective, with the filter sets: FITC, TRITC, and Cy5. Images were processed using DV SoftWoRX software. Fluorescence intensity calculations, corrected according to the area, were performed using Fiji (NIH, Bethesda, MD, USA). For the “APP colocalization” experiments, photomicrographs were acquired using the Zeiss LSM 880 confocal microscope (Carl Zeiss, Germany) equipped with a 63× oil immersion objective, with the filter sets: FITC, TRITC, and Cy5. Colocalization analyses of the photomicrographs were performed using the “Colocalization Analysis” plugin of Fiji (NIH, Bethesda, MD, USA), which provided the corresponding Mander’s overlap data.

For live-cell imaging experiments, movies were acquired using the Zeiss LSM 880 confocal microscope equipped with a climate chamber, wherein frames were acquired every 3 s for 180 s using a 63× oil immersion objective with a zoom of 1.5–2.5. Vesicle tracking, as well as vesicle fusion/fission analyses, were computed using a Fiji plugin—TrackMate (Tinevez et al., 2017). DoG detector of TrackMate was used for segmentation of RAB5-labeled vesicles, and the autothreshold setting of TrackMate was used to select only those vesicles whose fluorescence intensity was sufficiently high with respect to the background fluorescence intensity. Subsequently, the LAP tracker of TrackMate, which uses frame-to-frame linking for the construction of vesicle trajectories, was used for tracking vesicles and monitoring vesicle fusion/fission events. The following configuration was used for LAP tracker: maximum linking distance = 500 nm; maximum gap-closing distance = 500 nm; maximum frame gap = 2. Merging vesicle trajectories were considered as a vesicle fusion event, whereas a splitting vesicle trajectory was considered as a vesicle fission event. After configuration, TrackMate generates trajectories of the different vesicles in a cell and generates the appropriate data in a “heat-map” format to show the dynamic range of vesicle motility/displacement/fusion/fission as well as the corresponding raw data.

### Statistical Analyses

All data generated are from at least three independent experiments. The number of cells considered for statistics for each experiment is mentioned in the respective figure legend. Statistical analyses for all experiments were performed using the unpaired two-tailed Student’s *t*-test followed by the *post hoc* Bonferroni test, using the GraphPad Prism 6 software (GraphPad Software Inc., San Diego, CA, USA). Grubbs’ test was used to remove any significant outliers (*p* < 0.05) in the experimental data. Error bars represent mean ± SEM. A *p*-value of less than 0.05 was considered statistically significant.

## Results

### Expression of Markers Associated With Endolysosomal Dynamics Is Affected Upon 4R0N Tau Overexpression

**Figure 1:**
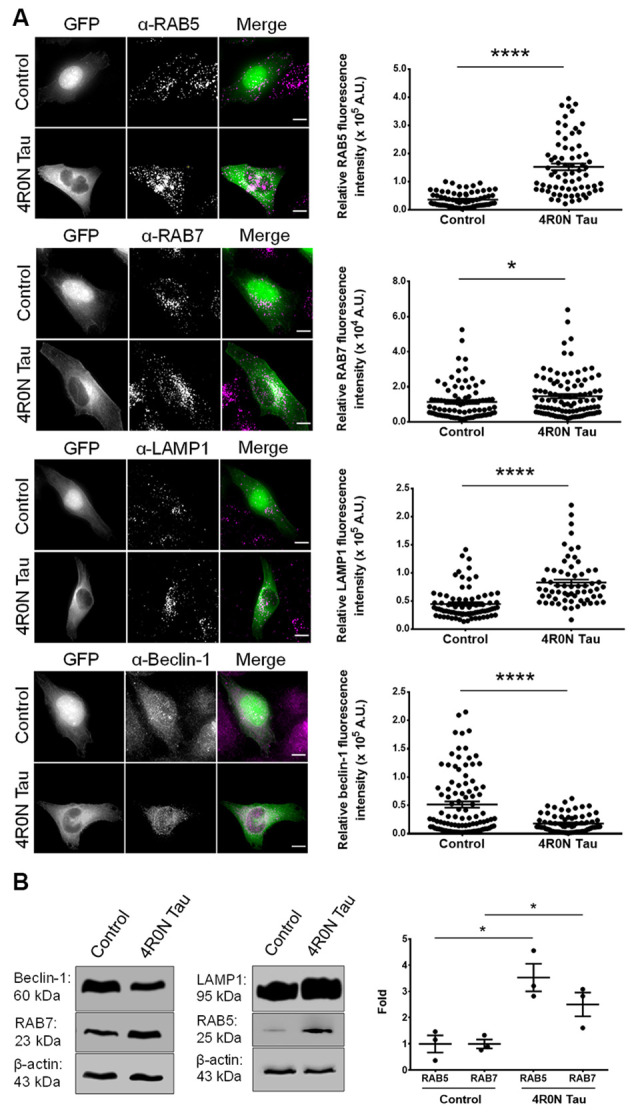
4R0N tau overexpression modulates the expression of markers associated with the endolysosomal system and autophagy. **(A)** Representative immunofluorescence maximum intensity projection photomicrographs of vector control and 4R0N tau-overexpressing HeLa cells immunoassayed for (starting from top) RAB5, RAB7, LAMP1, and beclin-1, and corresponding statistical analyses of relative fluorescence intensities. The signals of the antibody staining and the “GFP” channel are represented in magenta and green, respectively, in the “Merge” channel. Expression levels of RAB5, RAB7, LAMP1, and beclin-1 were found to be altered in the 4R0N tau-overexpressing cells. For quantification and statistical analysis, 12 *z*-stacks of individual cells were considered (*N* = 25–30 cells per independent experiment). The relative fluorescence intensity data displayed were corrected according to the corresponding relative GFP fluorescence intensity data as well as cell area. Scale bar: 10 μm. “A.U.”, arbitrary units. **(B)** Representative immunoblots showing the expression levels of RAB5, RAB7, LAMP1, and beclin-1 with statistical data for RAB5 and RAB7 expression levels. β-actin was used as the loading control, and further, the experimental data were corrected according to the average of the respective control densitometry values. Statistical analysis was performed using unpaired two-tailed Student’s *t*-test. Error bars reflect mean ± SEM, *p < 0.05, ****p < 0.0001. *n =* 3 independent experiments.

Prior to the investigation of endolysosomal system dynamics, we first elucidated the expression of specific markers involved in the endolysosomal pathway such as RAB5, RAB7, and LAMP1. Using immunofluorescence, we found that the expression levels of RAB5 (*p* < 0.0001), RAB7 (*p* < 0.05), and LAMP1 (*p* < 0.0001) were significantly upregulated in HeLa cells transiently transfected with 4R0N tau (pRK5-EGFP-tau) compared to HeLa cells transiently transfected with the vector control (pEGFP-N1). Additionally, we determined the expression of beclin-1, a key regulator of endocytic trafficking, early endosome maturation, and autophagy progression (Liang et al., 1999; Lucin et al., 2013; McKnight et al., 2014). The expression level of beclin-1 was found to be significantly downregulated (*p* < 0.0001) in the 4R0N tau-overexpressing cells, compared to those of the control (Figure 1A). These observations were corroborated by immunoblot experiments, which showed a similar trend in the expression levels of these markers (Figure 1B). Further investigation is required to understand some of the observed expression patterns.

### Increased RAB7 Expression Could be Attributed to an Increase in the Number of Autophagic Vacuoles in the Background of 4R0N Tau Overexpression

**Figure 2:**
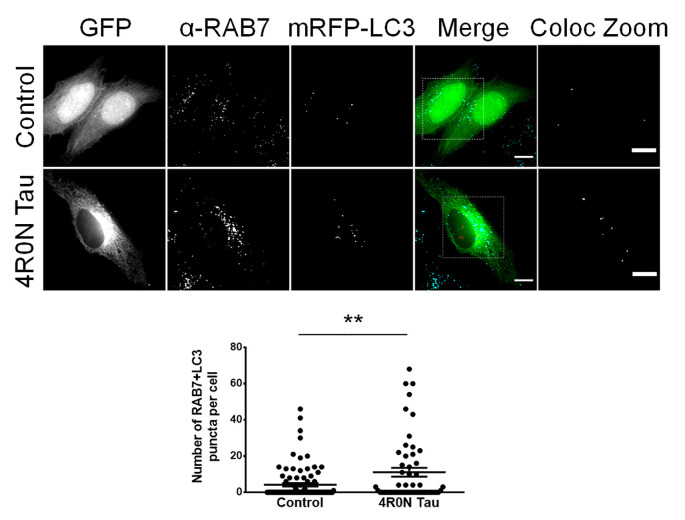
Increased number of autophagic vacuoles in HeLa cells overexpressing 4R0N tau. Representative immunofluorescence single *z*-stack photomicrographs of vector control and 4R0N tau-overexpressing cells cotransfected with the pmRFP-LC3 (mRFP-LC3) construct and immunostained for RAB7 and corresponding statistical data. The signals of the antibody staining, the “GFP” channel, and the “mRFP-LC3” channel are represented in cyan, green, and red, respectively, in the “Merge” channel. The insets in the “Merge” channel are magnified further, and only the RAB7+LC3 (colocalization of mRFP-LC3 puncta with RAB7 puncta) puncta, of both groups, are shown as the “Coloc Zoom” channel, representing the number of autophagic vacuoles. A significant increase in the number of autophagic vacuoles is observed in the 4R0N tau-overexpressing cells. For quantification and statistical analysis, three *z*-stacks of individual cells were considered (*N* = 20–30 cells per independent experiment). Statistical analysis was performed using unpaired two-tailed Student’s *t*-test. Scale bar: 10 μm. Error bars reflect mean ± SEM, **p < 0.01. *n =* 3 independent experiments.

Unlike RAB5, which typically labels early endosomes, RAB7 labels late endocytic compartments and lysosomes in addition to autophagic vacuoles. Autophagic vacuoles include autophagosomes, amphisomes, and autolysosomes, commonly labeled with the protein marker, LC3; autophagosomes and amphisomes ultimately mature into autolysosomes (Gutierrez et al., 2004; Jäger et al., 2004; Colacurcio et al., 2018). Hence, the RAB7 readout from the previous immunofluorescence experiment could indicate an increase in either the number of RAB7-labeled endocytic compartments or an increase in the number of RAB7+LC3-labeled autophagic vacuoles. To address this, we cotransfected cells of the control and 4R0N tau groups with pmRFP-LC3 and immunostained for RAB7 to examine the number of autophagic vacuoles. Through this experiment, we observed that the 4R0N tau-overexpressing HeLa cells displayed a significantly higher number of autophagic vacuoles (*p* < 0.01) compared to that of the control cells (Figure 2). Therefore, the initially observed increase in the expression of RAB7 could be indicative of an increase in the number of RAB7+LC3-labeled autophagic vacuoles instead of RAB7-labeled endocytic compartments. The elevated number of autophagic vacuoles in the 4R0N tau-overexpressing cells could be immature due to the reduced expression of beclin-1 observed earlier, potentially representative of autophagic stress induced by overexpression of 4R0N tau.

### 4R0N Tau Abrogates Maturation and Spatiotemporal Dynamics of Early Endocytic Vesicular Compartments

**Figure 3:**
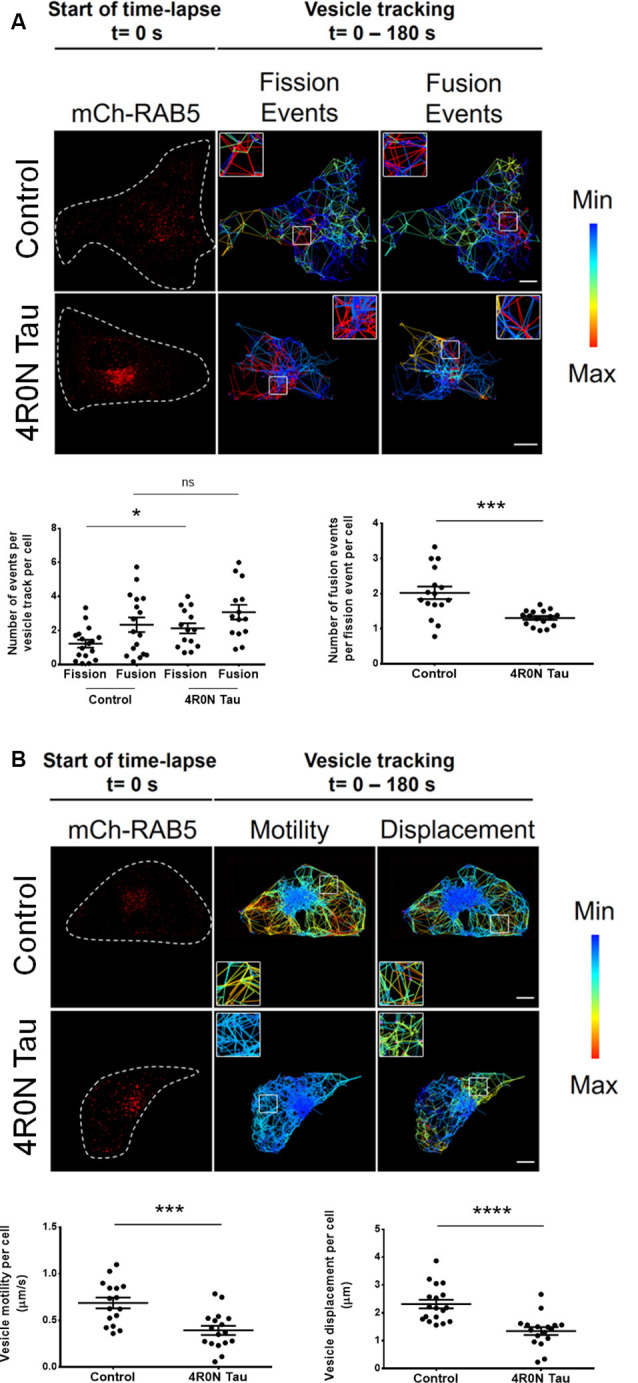
Live-cell imaging of mCh-RAB5-transfected HeLa cells shows the abrogation of early endosome vesicular dynamics in the background of 4R0N tau overexpression. The panels show the representative TrackMate-generated trajectory overlay of the time-lapse (single *z*-stack) of vector control and 4R0N tau-overexpressing HeLa cells coexpressing mCh-RAB5 (red) and corresponding statistical data. **(A)** Vesicular fission and fusion events are depicted as a heat map, as generated by TrackMate (blue represents minimum fission/fusion; red represents maximum fission/fusion). The insets that are shown in the “Fission Events” and “Fusion Events” channels are magnified and shown on the top left (Control) or top right (4R0N tau) corners of the respective channels. The frequency of vesicle fission events is increased in the 4R0N tau-overexpressing cells, while the frequency of vesicle fusion events does not show a concomitant increase, suggesting an impairment in early endosome maturation. The corresponding graphs depicting the number of fusion and fission events as well as the number of fusion events per fission event of both groups are shown below the panel. **(B)** The range of vesicle motility and displacement is depicted as a heat map, as generated by TrackMate (blue represents minimum motility/displacement; red represents maximum motility/displacement). The insets shown in the “Motility” and “Displacement” channels are magnified and shown in the bottom left (Control) or top left (4R0N tau) corners of the respective channels. The vesicle motility and displacement of RAB5-labeled vesicles are lower in the 4R0N tau-overexpressing cells. The corresponding graphs depicting the vesicle motility and displacement of both groups are shown below the panel. Vesicles in the 4R0N tau-overexpressing cells follow a random pattern unlike the well-defined pattern of the vesicles in the cells of the vector control group. The vesicular dynamics were tracked for 180 s with frames taken every 3 s. For quantification and statistical analysis, the middle *z*-stack of individual cells was considered (*N* = 14–19 cells). White dashed lines demarcate individual cells. Statistical analysis was performed using unpaired two-tailed Student’s *t*-test. Scale bar: 10 μm. Error bars reflect mean ± SEM, *p < 0.05, ***p < 0.001, ****p < 0.0001, ns, not significant. *n* = 3 independent experiments.

It is unclear whether the previously observed increase in RAB5 expression is indicative of upregulated or disrupted endocytic flux. To address this, we performed live-cell imaging and vesicular tracking analyses (refer to the “Materials and Methods” section, for more details). It is well known that the homotypic fusion of early endosomes is necessary for their maturation into late endosomes. Consequently, we hypothesized that if endocytic flux was negatively impacted at the maturation stage of early endosomes, then the ratio of vesicle fusion events to vesicle fission events would be altered. To test this hypothesis, live-cell imaging of vector control and 4R0N tau-overexpressing HeLa cells cotransfected with mCh-RAB5 was conducted, to evaluate the number of vesicle fusion and fission events. The number of RAB5-labeled vesicle fission events (*p* < 0.05) was found to be elevated in 4R0N tau-overexpressing cells compared to that of the control cells, while the number of RAB5-labeled fusion events did not significantly differ in both groups of cells (Figure 3A). Additionally, the ratio of vesicle fusion events to vesicle fission events was significantly less (*p* < 0.001) in the 4R0N tau-overexpressing cells compared to that of the control cells (Figure 3A). These observations hint toward an inherent bias for RAB5-labeled vesicle fission in the 4R0N tau-overexpressing cells. To further understand the differences between the groups, we studied the spatiotemporal aspects of RAB5-labeled vesicles. Overall motility (*p* < 0.001) and displacement (*p* < 0.0001) of RAB5-labeled vesicles were found to be dramatically reduced under 4R0N tau overexpression (Figure 3B). Moreover, the trajectories of the vesicles followed a random pattern compared to that of the vector control cells (Figure 3B), which showed clearly defined trajectories. These data indicate that 4R0N tau hampers the maturation of RAB5-labeled early endocytic compartments and impedes the trafficking of these vesicles. These observations suggest that the previously observed upregulation of RAB5 expression could stem from the disruption of endocytic flux at the early endocytic stage, attributable to 4R0N tau overexpression.

### 4R0N Tau Overexpression Promotes the Sequestration of APP Into Endocytic Compartments

**Figure 4:**
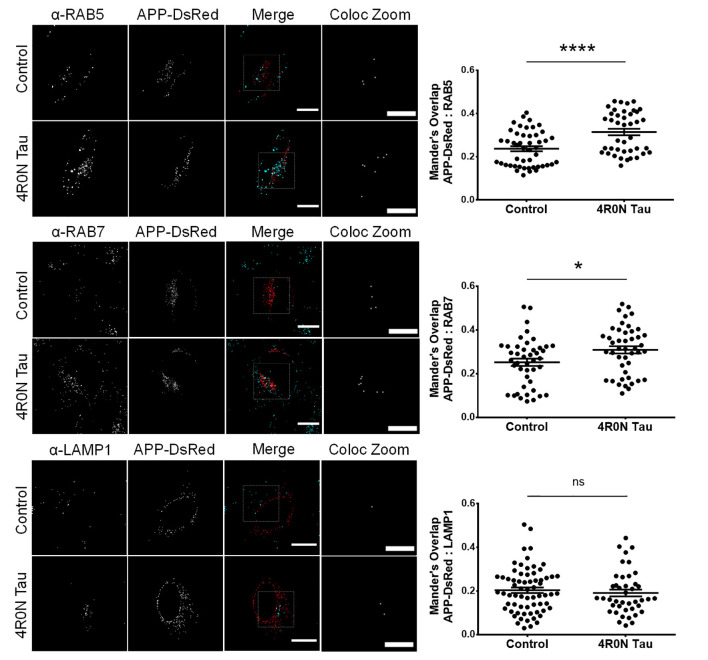
4R0N tau overexpression promotes the sequestration of amyloid precursor protein (APP) into the endocytic compartments. Representative immunofluorescence single *z*-stack photomicrographs of pDsRedm-C1:APP (APP-DsRed)-transfected HeLa cells in the background of vector control or 4R0N tau overexpression and immunostained for (starting from top) RAB5, RAB7, and LAMP1, and corresponding Mander’s overlap statistical data. The signals of the antibody staining and the “APP-DsRed” channel are represented in cyan and red, respectively, in the “Merge” channel. The “Coloc Zoom” channel of the panel represents a magnified view of the corresponding insets in the “Merge” channel, to show the colocalization of APP-DsRed puncta with puncta of the respective endolysosomal marker. An increased amount of APP colocalizes with RAB5- and RAB7-labeled vesicles, but not with LAMP1-labeled vesicles, in the 4R0N tau-overexpressing cells, indicating increased susceptibility of APP to undergo BACE1-mediated cleavage, the rate-limiting step of the amyloidogenic pathway. For quantification and statistical analysis, three *z*-stacks of individual cells were considered (*N* = 15–25 cells per independent experiment). Statistical analysis was performed using unpaired two-tailed Student’s *t*-test. Scale bar: 10 μm. Error bars reflect mean ± SEM, *p < 0.05, ****p < 0.0001, ns, not significant. *n =* 3 independent experiments.

From the previous experiments, we observed that the endocytic vesicular dynamics were affected due to 4R0N tau overexpression. As a consequence, we hypothesized that the trafficking of specific endocytic cargoes could also be affected. Of the many endocytic cargoes, APP piqued our interest due to its implications in AD. In order to elucidate the degree of APP colocalization with markers of the endocytic pathway, we cotransfected the control and 4R0N tau-overexpressing cells with the pDsRedm-C1:APP construct, which encodes APP with a C-terminal DsRed tag. These cells were then immunostained to detect the expression of the respective endolysosomal protein marker. The colocalization channel was used to evaluate the degree of APP colocalization along the endocytic pathway (refer to the Materials and Methods section, for more details). In the 4R0N tau-overexpressing cells, APP was selectively sequestered to RAB5-labeled (*p* < 0.0001) and RAB7-labeled (*p* < 0.05) vesicles (Figure 4). There was no significant difference in the colocalization of APP with LAMP1 (ns; Figure 4) between the two groups of cells. These observations indicate that tau overexpression disrupts the trafficking of APP such that APP accumulates in the endocytic compartments contributing to amyloidogenic processing instead of being delivered to the lysosomal compartments for degradation.

## Discussion

Our investigation revealed that the dynamics of the endolysosomal system and autophagy are compromised upon overexpression of soluble 4R0N tau. We observed that the expression levels of RAB5, RAB7, and LAMP1 were affected due to soluble 4R0N tau overexpression. These results are in agreement with the AD clinical studies wherein a similar expression profile of these markers was observed (Ginsberg et al., 2010a,b, 2011). Similarly, APP/Aβ-related pathology has also been found to positively regulate the expression levels of RAB5, RAB7, and LAMP1, aside from affecting associated endocytic vesicle morphology (Ji et al., 2006; Cataldo et al., 2008; Kim et al., 2016; Willén et al., 2017). Thus, the primary causative factor responsible for such an expression profile in AD clinical cases remains yet to be identified, but it is worth noting that both tau and Aβ-related pathologies can individually affect the endolysosomal system in similar ways. Studies have reported that RAB5- and RAB7-labeled vesicles are the primary sites of amyloidogenesis in a cell (Haass et al., 2012; Das et al., 2015). Enhanced expression of these RABs could indicate a greater propensity for Aβ production due to an increased pool of endocytic APP resulting in increased amyloidogenesis (Grbovic et al., 2003). We also found that soluble 4R0N tau overexpression leads to decreased beclin-1 expression, which is in agreement with AD-related clinical studies (Rohn et al., 2011; Orr and Oddo, 2013). Beclin-1 regulates autophagy, as well as endocytic trafficking and maturation through its ability to recruit the retromer complex to the endosomal compartments (Liang et al., 1999; Lucin et al., 2013; McKnight et al., 2014). Additionally, it has been shown that beclin-1 can directly regulate APP endocytosis (Swaminathan et al., 2016). Therefore, a deficiency of beclin-1 could potentially impair endocytic trafficking and maturation leading to increased accumulation of APP in the endocytic compartments, thereby increasing the susceptibility of APP to undergo amyloidogenic processing. Furthermore, we observed an increased number of autophagic vacuoles, possibly immature, in the soluble 4R0N tau-overexpressing cells, which could be due to autophagic stress in correlation to beclin-1 deficiency. A similar phenotype has been noted in AD patient brains, deficient of beclin-1, wherein autophagic vacuoles were shown to be accumulated in dystrophic neurites, unable to fuse with functional lysosomes (Nixon et al., 2005; Li et al., 2010; Tammineni et al., 2017). The observed down-regulation of beclin-1 was concluded to be independent of Aβ pathology since APP transgenic mouse models of AD, based on Aβ pathology, did not exhibit decreased beclin-1 expression levels (Pickford et al., 2008). Overall, these observations support the hypothesis that the pathological effects of tau precede Aβ-associated pathology, with beclin-1 deficiency acting as an intermediary factor responsible for the dysregulation of endolysosomal dynamics and autophagy. Our observations outline a framework in agreement with the outlook of AD-related clinical studies.

The previous conclusion is further strengthened by the colocalization experiments, which aimed to elucidate the localization of APP upon soluble 4R0N tau overexpression. We found that APP was selectively sequestered to RAB5- and RAB7-labeled vesicles. Disruption of endocytic flux is evident since a concomitant increase in the colocalization of APP with LAMP1-labeled lysosomes was not observed. Further, the impaired maturation and trafficking of RAB5-labeled vesicles observed in our study could suggest delayed turnover of early endocytic compartments, which can, in turn, influence the rate of amyloidogenesis since APP is not able to reach the lysosomes for proteolytic degradation. Collectively, the delicate balance between the degradation and amyloidogenic processing of APP is affected due to soluble 4R0N tau overexpression. This leads us to postulate that soluble 4R0N tau pathology could underscore AD pathogenesis through disruption of endolysosomal function, autophagic stress, and enhanced amyloidogenic processing of APP at endocytic compartments, although further research is warranted. However, it must be noted that the mechanistic link between soluble tau overexpression and the reported phenotypes remains elusive, and it should be an important subject for future studies. Based on previous literature, it is plausible that soluble tau could localize in the lumen and/or the membrane of endocytic vesicles (Vaz-Silva et al., 2018), and interact with ESCRT machinery-associated proteins potentially leading to dysfunction of the ESCRT pathway, which is associated with impaired endosomal trafficking and function (Watson et al., 2015; Oshima et al., 2016). Further experimentation is required to unequivocally establish the mode of soluble tau pathology that causes the phenotypes observed in our study, owing to soluble 4R0N tau overexpression. Furthermore, it is important to note that the observations reported in our study must be validated with other appropriate models, and hence, it must be interpreted with caution.

The model proposed by our study is in line with documented AD clinical observations. Biomarker studies based on cerebrospinal fluid (CSF) and neuropathological analysis of AD clinical cases have revealed a specific temporal pattern of accumulation of pathological proteins. To substantiate, while it has been shown that reduction of Aβ protein levels in the CSF precedes that of tau aggregates by many years, the accumulation of tau with AD relevant phosphorylation epitopes takes place decades before Aβ plaque formation (Motter et al., 1995; Braak et al., 2011, 2013; Musiek and Holtzman, 2012). Moreover, fibrillary Aβ and tau pathologies were found to be spatiotemporally disconnected within the human central nervous system (Thal et al., 2000; Braak et al., 2006). Such a disconnect between the fibrillary versions of the two critical biomarkers of AD, Aβ and tau, makes it unclear as to which biomarker of AD takes precedence. More importantly, it questions the very idea of a physiological cross-talk between Aβ and tau that leads to pathology. Contrastingly, the soluble forms of tau and Aβ show a much better overlap of neuropathological association in the temporal lobe of the AD brain, and this cross-talk event occurs before their terminal state as fibrillary counterparts are observed in a similar location (Koss et al., 2016). Therefore, from a clinical standpoint, it has been demonstrated that the soluble forms of Aβ and tau can cross-talk prior to aggregation. This critical period, before the nucleation of aggregates, requires further investigation to firmly establish the sequence of events that connect the pathologies of Aβ and tau. In this regard, our study has found a possible mode of cross-talk between tau and Aβ.

To conclude, our study has revealed the pathological profile of soluble 4R0N tau overexpression in cells, which recapitulates some of the earliest phenotypes observed in AD clinical cases such as endolysosomal dysfunction and autophagic stress (Orr and Oddo, 2013; Whyte et al., 2017; Colacurcio et al., 2018). The impaired dynamics of the endolysosomal system have serious implications in the generation of Aβ. With several AD clinical trials targeting aggregated tau, our findings enforce the notion that therapeutic intervention must be focused on soluble tau pathology.

## Data Availability Statement

All datasets generated for this study are included in the article.

## Author Contributions

RM, SS, and TM designed the study. LG provided technical support and helped with the cell culture experiments. SS and TM analyzed the data. RM, SS, and TM interpreted the findings of the study. TM performed the experiments and prepared the final manuscript, which was critically reviewed by all the other authors. All authors contributed to the article and approved the submitted version.

## Conflict of Interest

The authors declare that the research was conducted in the absence of any commercial or financial relationships that could be construed as a potential conflict of interest.
